# Comparative Assessment of Different Ultrasound Technologies in the Detection of Prostate Cancer: A Systematic Review and Meta-Analysis

**DOI:** 10.3390/cancers15164105

**Published:** 2023-08-15

**Authors:** Dareen Alghamdi, Neil Kernohan, Chunhui Li, Ghulam Nabi

**Affiliations:** 1 Division of Imaging Sciences and Technology, School of Medicine, Ninewells Hospital, University of Dundee, Dundee DD1 9SY, UK; 2 Radiology Department, College of Applied Medical Sciences, Imam Abdulrahman Bin Faisal University, P.O. Box 1982, Dammam 31441, Saudi Arabia; 3 Department of Pathology, Ninewells Hospital, Dundee DD9 1SY, UK; n.m.kernohan@dundee.ac.uk; 4 School of Science and Engineering, University of Dundee, Dundee DD1 4HN, UK; c.li@dundee.ac.uk

**Keywords:** prostate cancer, PSA, micro-ultrasound, multiparametric ultrasound, grayscale, shear wave elastography, contrast-enhanced ultrasound

## Abstract

**Simple Summary:**

Prostate cancer (PCa) is considered as one of main causes of death in men globally. More research is required on the diagnostic accuracy of mpUS and advanced modalities in prostate cancer detection, which may provide insightful information into the diagnostic accuracy and clinical utility of this technique. Therefore, we have conducted a systematic review and meta-analysis to assess the diagnostic test accuracy of different ultrasound scanning technologies (shear-wave elastography, contrast enhanced, micro-ultrasound) and grayscale ultrasound technology in the detection of prostate cancer. This will assist in determining whether this new method of detecting prostate cancer is effective. Our results showed that some studies proved that advanced ultrasound modalities are promising methods for the detection of prostate cancer.

**Abstract:**

The present study aimed to assess the diagnostic test accuracy of different ultrasound scanning technologies in the detection of prostate cancer. A systematic search was conducted using the Cochrane Guidelines for Screening and Diagnostic Tests. We performed a systematic search in the international databases PubMed, Medline, Ovid, Embase and Cochrane Library. Searches were designed to find all studies that evaluated Micro-US, mpUS, SWE and CEUS as the main detection modalities for prostate cancer. This study was registered with Research Registry of systematic review and meta-analysis. The QUADAS-2 tool was utilized to perform quality assessment and bias analysis. The literature search generated 1376 studies. Of these, 320 studies were screened for eligibility, with 1056 studies being excluded. Overall, 26 studies with a total of 6370 patients met the inclusion criteria. The pooled sensitivity for grayscale, CEUS, SWE, Micro-US and mpUS modalities were 0.66 (95% CI 0.54–0.73) 0.73 (95% CI 0.58–0.88), 0.82 (95% CI 0.75–0.90), 0.85 (95% CI 0.76–0.94) and 0.87 (95% CI 0.71–1.03), respectively. Moreover, the pooled specificity for grayscale, CEUS, SWE, Micro-US and mpUS modalities were 0.56 (95% CI 0.21–0.90), 0.78 (95% CI 0.67–0.88), 0.76 (95% CI 0.65–0.88), 0.43 (95% CI 0.28–0.59) and 0.68 (95% CI 0.54–0.81), respectively. In terms of sensitivity, substantial heterogeneity between studies was detected (I^2^ = 72%, *p* = 0.000 < 0.05). In relation to specificity, extreme heterogeneity was detected (I^2^ = 93%, *p* = 0.000 < 0.05). Some studies proved that advanced ultrasound modalities such as mpUS, Micro-US, shear-wave elastography, contrast enhanced and micro-ultrasound are promising methods for the detection of prostate cancer.

## 1. Introduction

Prostate cancer (PCa) is considered as one of the main causes of death in men globally [1]. Prostate cancer can be discovered using a variety of methods, including measuring prostate specific antigen (PSA) levels, digital rectal examination (DRE) and conventional TRUS biopsy [2,3]. According to Lumbreras et al. [4], PSA has a high false-positive rate, causing many useless systematic biopsies. Moreover, it has been reported that the DRE will not greatly decrease death rates; rather, it can produce a large number of false positives, resulting in redundant aggressive diagnostic tests which cause pain, sexual dysfunction, bladder problems, misdiagnosis and overtreatment of prostate cancer [2,5]. Furthermore, there is a growing recognition that the TRUS systemic biopsy method, which uses random sampling, may miss csPCa. It has also been reported that the conventional TRUS ultrasound has a high false-negative rate [3,6]. Consequently, other methods are being explored. For instance, magnetic resonance imaging (MRI) is presently the most effective method for detecting prostate malignant tumors. Nevertheless, there are some limitations to using MRI alone [7,8,9]. Thus, multiparameter magnetic resonance imaging (mp-MRI) is a more effective method used in diagnosing csPCa [7,10]. However, mpMRI is too expensive and time-consuming, and some contraindications (claustrophobia, pacemaker, etc.) suggest that it has a primary diagnostic modality [3,11].

Grayscale (GS) TRUS is one of the most frequently used imaging techniques for direct visualization of the prostate due to its real-time function, low radiation and relatively low cost [12,13]. Traditional grayscale TRUS is thought to have a partial role in PCa detection [14]. Several studies reported that the sensitivity of TRUS grayscale ranged between 11 and 35% and its positive predictive value (PPV) ranged between 27 and 57% [15]. Thus, its benefits have largely driven the advancement of innovative ultrasound modalities aimed at increasing PCa detection, such as contrast-enhanced ultrasound (CEUS), computerized TRUS, and (shear-wave) elastography [11,16]. However, due to various types of enhanced micro-vascularity and a stromal reaction that results in increased collagen deposition around the tumor, prostate cancers are more difficult to treat than normal prostatic tissue [17]. Thus, elastography is another effective modality used to assess tissue rigidity rather than echogenicity, providing an innovative technique for identifying pathological abnormalities that would otherwise go undetected by conventional ultrasound (US) [18]. SWE is therefore considered a novel technique for measuring tissue stiffness at the local level. SWE is based on measuring shear-wave velocity as it propagates through tissue without the requirement for manual compression. This method offers numerical evidence of tissue rigidity in the form of Young’s modulus (kPa) [19]. However, SWE has significant drawbacks: some types of cancers are not rigid and other cancerous lesions are not stiff (calcification and fibrosis) [18]. As a result, the diagnostic value of SWE on its own is still debatable [20].

Hence, the new method of contrast-enhanced ultrasound (CEUS) is being currently applied to differentiate some lesions that are hard to see [21]. The CEUS is thought to improve the visibility of focal lesions in organs [21,22,23]. Therefore, as a novel imaging technique, contrast-enhanced ultrasound (CEUS) may dynamically recognize the blood perfusion of vascularity, particularly feeding micro neovascularity linked with tumor. Even in the early stages of PCa, angiogenesis causes increased flow, and CEUS can show imbalance of intraprostatic vessels and focal advancement [22,24]. Even though CEUS is a valid technique due to its own unique benefits, guidelines do not recommend it as a regular method for merging with MRI for the diagnosis of PCa, due to its defined and device-dependent interobserver accuracy shortcomings [25,26]. In addition, micro ultrasound (Micro-US) is another modality used to detect PCs. It is one of the latest modalities that uses a high frequency (29 MHz) to produce images with a resolution of approximately 70 µm [20]. The Micro-US operation is almost indistinguishable from the traditional TRUS procedure, with the added advantage of improved image resolution and visualization of suspicious tissue, allowing for real-time targeted biopsies. Previous research suggested that Micro-US assists in screening procedures by guaranteeing that men with PCa are offered a biopsy as soon as possible [20,27].

The most recent systematic review of the improvements and clinical outcomes of various ultrasound modalities was published by Postema et al. [14] in 2015. Their study investigated the progress and clinical performance of various ultrasound modalities, including the development of combining such modalities with multiparametric ultrasound (mpUS). The mpUS method refers to a combination of various ultrasound examinations, such as TRES, TRUS grayscale and CEUS [14,15]. It was reported that the (mpUS) method could potentially decrease the possibility of missing tumors that were not noticeable with one of the modalities and distinguish benign prostatic diseases such as prostatitis, which can mimic malignant characteristics. Postema et al. [14] showed that combining ultrasound modalities enhanced diagnostic performance significantly. However, their study provided little information about their methods, for instance, it was not stated whether they included studies that assessed PSA in symptomatic or asymptomatic patients. In addition, a meta-analysis to resolve conflicts between studies and produce conclusive results was not conducted.

Therefore, the present study focused on systematic synthesis of the reported literature on different ultrasound modalities in the detection of prostate cancer and identified gaps in the literature and areas for future research to improve prostate cancer detection and diagnosis using mpUS. Specifically, we assessed the following:

(1) Diagnostic accuracy of transrectal SWE ultrasound in the detection of prostate cancer.
(2) Diagnostic accuracy of CEUS in the detection of prostate cancer.
(3) Diagnostic accuracy of micro-ultrasound in the detection of prostate cancer.
(4) Diagnostic accuracy of multiparametric ultrasound in the detection of prostate cancer.

## 2. Materials and Methods

### 2.1. Search Strategy and Selection Criteria

A systematic review and meta-analysis were conducted by using the Cochrane Guidelines for Screening and Diagnostic Tests. Eligible studies mainly included published peer-reviewed articles between 2018 and 2023. According to the PRISMA guidelines, we performed a systematic search in the international databases PubMed, Medline, Ovid, Embase and Cochrane Library [28]. Searches were designed to find all studies that evaluated Micro-US, mpUS, grayscale, elastography and CEUS as the main detection modalities for prostate cancer, both in terms of screening and diagnostically. This study was registered with the Research Registry for the systematic review and meta-analysis (Review Registry ID: reviewregistry1660). Moreover, a detailed search strategy that included MeSH terms was established. The search terms included the following: (prostate or prostatic) AND (cancer or carcinoma or neoplasm or malignancy or tumor) AND (evaluation, diagnosis, (sensitivity, specificity), or detection) AND (biopsy or pathology or histopathology) AND (prostate cancer) or (Micro-US) OR (mpUS) OR (grayscale) OR (elastography) OR (CEUS). Each relevant article’s reference list was also examined. In addition, gray literature, including reports and conference presentations, was reviewed.

The reference lists of the obtained articles were also investigated for further relevant articles. Inclusion and exclusion criteria were discussed and agreed among the authors. Inclusion criteria for this systematic review and meta-analysis included peer-reviewed studies with male participants that evaluated one of the following modalities: (Mciro-US, mpUS, grayscale, elastography and CEUS) as a detection modality for prostate cancer. Studies not written in English were excluded. Studies that targeted male patients (all ages) with a suspicion of PCs based on the elevated serum PSA concentration or abnormal digital rectal examination. The prostate-specific antigen (PSA), measured in nanograms per milliliter (ng/mL), was the index test for this review. Instead of establishing an a priori PSA threshold, we gathered information based on the PSA thresholds applied in every study. Moreover, the target condition was prostate cancer, while the reference was biopsy of the prostate cancer or radical prostatectomy, which are considered a histological examination.

Furthermore, the included studies comprise cross-sectional cohort studies, prospective and retrospective studies, in vivo studies, randomized and non-randomized studies and clinical trials. Moreover, we only included studies that reported the following outcomes: sensitivity, specificity, positive predictive value (PPV) and negative predictive value (NPV). We restricted studies by publication date: only studies that were published between January 2018 and January 2023 were included. However, we did not restrict studies based on country or clinical setting. A systematic review’s relevance and accuracy are maintained by incorporating studies from the last five years, contextualizing new findings within the body of knowledge and keeping the review up to date with advancements in the field of interest. Inclusion and exclusion criteria are shown in Table 1.

### 2.2. Data Extraction

The following data were extracted from the selected studies: name of authors, the year of publication, country, study design, target population, modality used, biopsies, outcome measure and study conclusion, number of patients, mean age (range), study setting and PSA ng/m range. The following data were also extracted from each study: sensitivity, specificity, PPV and NPV for the detection of prostate cancer.

### 2.3. Quality Assessment

The Quality Assessment of Diagnostic Accuracy Studies (QUADAS-2) tool was utilized to perform quality assessment and bias analysis [29]. Patient selection, index tests, reference standard, flow and timing, and applicability were all evaluated. Reviewers individually evaluated the quality of selected papers; overall results were based on agreement. QUADAS risk assessment results are represented. Overall, QUADAS-2 offers a structured and transparent method for evaluating the reliability of diagnostic accuracy studies. The quality assessment was conducted independently by one reviewer and checked by a second reviewer. Any disagreements were discussed among the reviewers. Review Manager (RevMan) version 5.4 was used to complete the QUADAS quality assessment.

### 2.4. Data Analyses

R 4.3.0 (2023) was used to assess the diagnostic performance of all modalities. The results that were extracted from all selected studies were grouped to generate summary estimations of sensitivity and specificity for PC detection. Moreover, from all the studies, a forest plot for combined sensitivity and specificity was created. In addition, a forest plot for sensitivity and specificity was created for each modality. Heterogeneity was assessed visually, using Forest plots of sensitivity and specificity. The I-squared was utilized to determine the heterogeneity between studies, and I 2 > 50% and P 0.1 indicated statistically significant heterogeneity. A funnel plot was also created to determine publication bias.

## 3. Results

### 3.1. Literature Search and Study Selection

**Figure 1:**
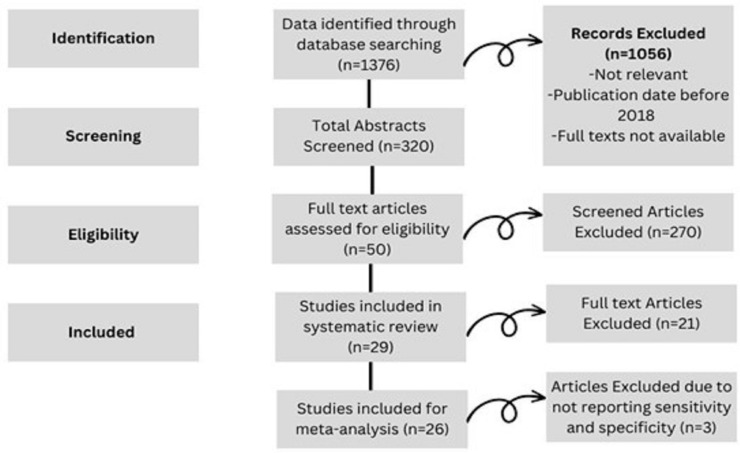
Prisma Flow Diagram.

The literature search with PubMed, Medline, Ovid, EMBASE and Cochrane Library generated 1376 searches. Of these, 320 studies were screened for eligibility, with 1056 studies being excluded. The excluded studies were inconsequential to the review aim: the publication date was before 2018 or the full texts were unavailable. Fifty full-text articles were reviewed for eligibility. Twenty-one of these studies were excluded as they lacked data on the targeted modalities, focused on alternative ultrasound modalities and/or did not report diagnostic accuracy of the targeted modalities. In addition, the studies lacked data on sensitivity and specificity. Overall, 26 studies were included in the final systematic review and meta-analysis (Figure 1).

### 3.2. Study Characteristics

The 26 included studies for the meta-analysis featured 6370 patients. Table 2 presents the technical characteristics of the patients who participated in the selected studies. The age ranged between 62 and 70 years old, and the PSA ranged from 1.09 to 60.83 ng/mL. Of the included studies, one study did not provide mean age [9] and two studies did not provide median PSA [9,30]. The selected studies were not relatively geographically diverse, as they only represented a total of nine countries. This included 10 studies in China, 7 in Italy, 4 in the United States, 2 in Germany, 2 in Korea, 1 in Japan and 1 in France. Regarding study settings, most studies were conducted in a single institute setting; only two studies were conducted in a multi-institute setting. The study characteristics for each modality are represented in Table 3 and Tables S1–S4. Of the 26 selected studies, 17 were conducted prospectively and 9 were conducted retrospectively. The inclusion criteria for the studies were either patients with a clinical suspicion of PC or patients with an elevated or increasing PSA. Moreover, 11 studies were performed with Micro-US [31,32,33,34,35,36,37,38,39,40,41], 2 studies with mpUS [30,42], 4 studies with grayscale [30,43,44,45], 7 studies with SWE [30,46,47,48,49,50,51] and 5 studies with CEUS [9,30,52,53,54].

### 3.3. Quality Assessment

**Figure 2:**
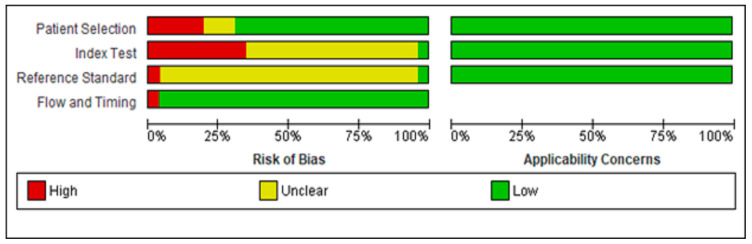
Risk of bias and applicability concerns graph: review authors’ judgments about each domain presented as percentages across included studies.

**Figure 3:**
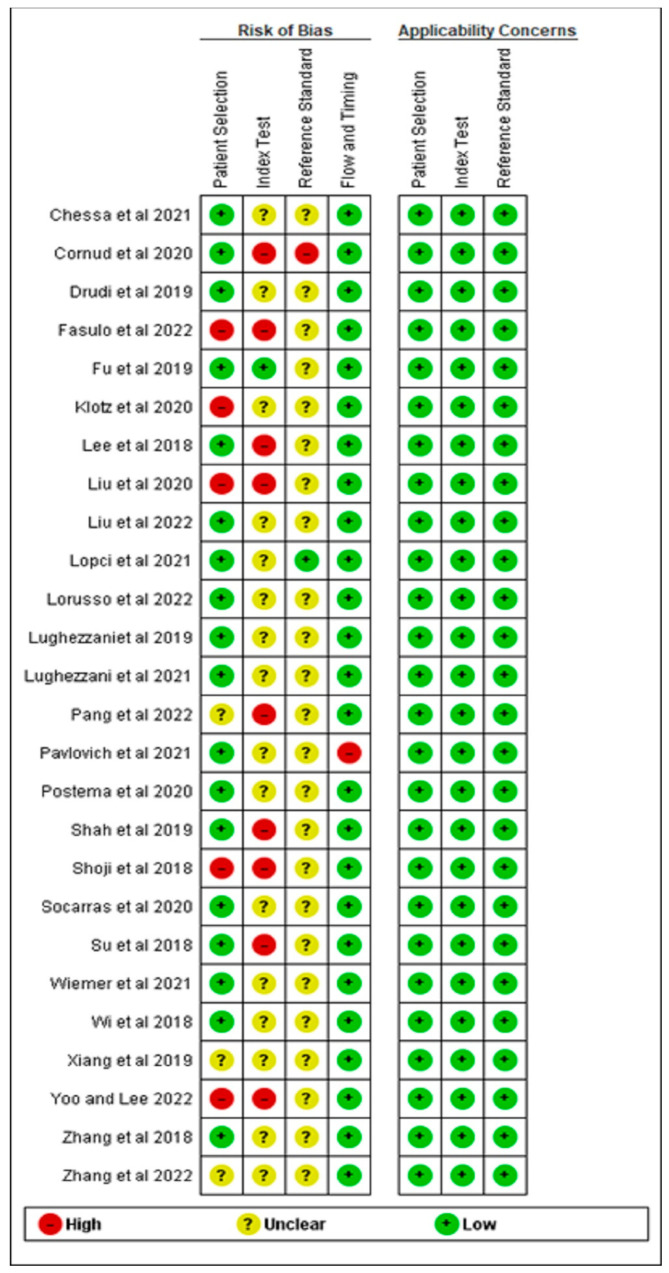
Risk of bias and applicability concerns summary: review authors’ judgements about each domain for each included study [9,30,31,32,33,34,35,36,37,38,39,40,41,42,43,44,45,46,47,48,49,50,51,52,53,54].

Overall, the quality of the studies was considered low risk (Figure 2 and Figure 3). For the patient selection domain, the majority of included studies had a low risk of bias with no inappropriate inclusion or exclusion criteria. However, five of the selected studies were considered high risk as they were not conducted randomly or consecutively [34,35,43,44,48]. In addition, three of the studies were unclear about how they selected the patients. Overall, all the studies were assigned low concerns regarding applicability [9,42,47].

For the index test domain, the majority of the selected studies were unclear of whether the index test (PSA ng/mL) results were interpreted without knowledge of the results of the reference standard (biopsy). Furthermore, none of the studies specified the threshold used. For the reference standard domain, most studies were unclear about whether the reference standard results were interpreted without knowledge of the results of the index tests. Overall, all the studies were assigned low concerns regarding applicability.

Only two studies [30,33] had a possible risk of bias in the flow and timing domain as they were unclear about whether there was an appropriate interval between index test and reference standard. In addition, the same reference standard was not used for all patients and not all patients were included in the analysis.

### 3.4. Sensitivity and Specificity Analysis

**Figure 4:**
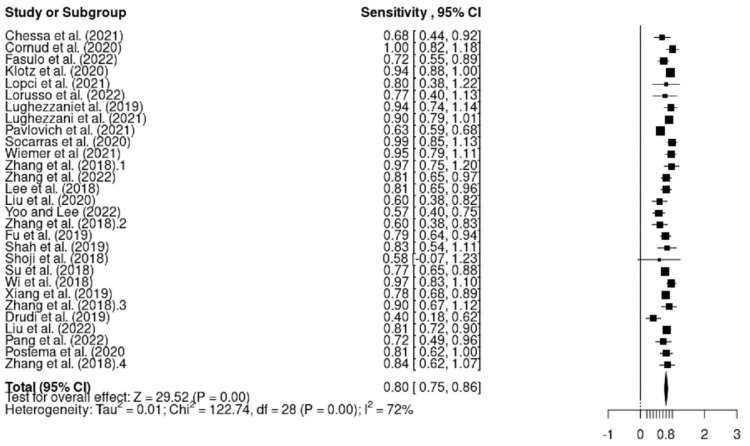
Pooled sensitivity for all modalities combined [9,30,31,32,33,34,35,36,37,38,39,40,41,42,43,44,45,46,47,48,49,50,51,52,53,54].

**Figure 5:**
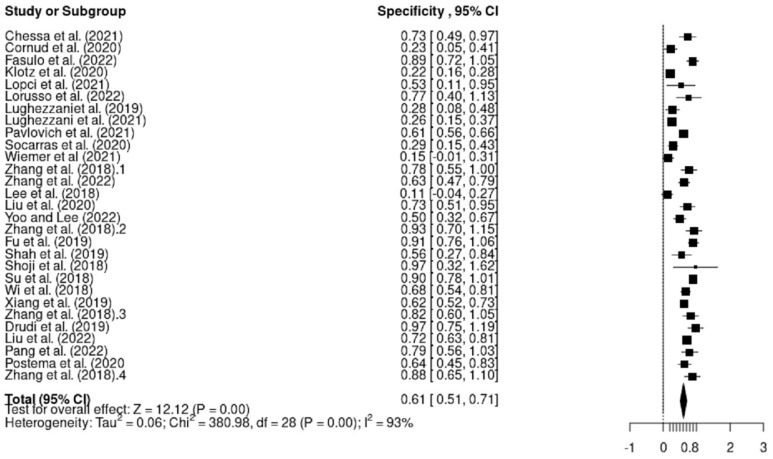
Pooled specificity for all modalities combined [9,30,31,32,33,34,35,36,37,38,39,40,41,42,43,44,45,46,47,48,49,50,51,52,53,54].

The pooled sensitivity and specificity for all studies combined was 0.80 (95% CI 0.75–0.86) and 0.61 (95% CI 0.51–0.71), respectively (Figure 4 and Figure 5).

From the 11 studies reporting findings on the use of Micro-US as a detection modality for prostate cancer, the sensitivity, specificity, positive predictive values (PPV) and negative predictive values (NPV) ranged from 0.68 to 1, 0.22 to 0.92, 0.35 to 0.93 and 0.31 to 0.96, respectively. Moreover, the pooled sensitivity and specificity for the Micro-US were 0.85 (95% CI 0.76–0.94) and 0.43 (95% CI 0.28–0.59), respectively (Figures S1 and S2). Furthermore, of the two studies [30,42] that assessed the performance of the mpUS, the sensitivity, specificity, PPV and NPV ranged from 0.81 to 0.97, 0.63 to 0.78, 0.70 to 0.90 and 0.71 to 0.97, respectively. The pooled sensitivity and specificity for the mpUS were 0.87 (95% CI 0.71–1.03) and 0.68 (95% CI 0.54–0.81), respectively (Figures S3 and S4). In addition, among the four studies reporting findings on the use of grayscale as a detection modality for prostate cancer, the sensitivity and specificity ranged from 0.58 to 0.81 and 0.11 to 0.93, respectively. However, only two of the studies reported the PPV and NPV of grayscale. Zhang et al. (2019) included 78 patients with an elevated or increasing PSA level (>4.0 ng/mL) and reported PPV and NPV of 0.89 and 0.71, respectively. Moreover, Lee et al.’s (2018) study on 157 patients reported the PPV and NPV as 0.47 and 0.38, respectively. The pooled sensitivity and specificity for grayscale were 0.66 (95% CI 0.54–0.73) and 0.56 (95% CI 0.21–0.90), respectively (Figures S5 and S6). Regarding the seven studies that reported elastography as a detection modality for prostate cancer, the sensitivity, specificity, PPV and NPV ranged from 0.58 to 0.97, 0.56 to 0.97, 0.47 to 0.86 and 0.79 to 0.89, respectively. The pooled sensitivity and specificity for shear-wave elastography were 0.82 (95% CI 0.75–0.90) and 0.76 (95% CI 0.65–0.88), respectively (Figures S7 and S8). Lastly, from the five studies reporting findings on the use of contrast-enhanced ultrasound (CEUS) as a detection modality for prostate cancer, the sensitivity, specificity, PPV and NPV ranged from 0.40 to 0.84, 0.64 to 0.97, 0.87 to 0.97 and 0.55 to 0.92, respectively. However, PPV and NPV were not reported by Pang et al. [9] or Postema et al. [54]. Moreover, the pooled sensitivity and specificity for the CEUS were 0.73 (95% CI 0.58–0.88) and 0.78 (95% CI 0.67–0.88), respectively (Figures S9 and S10).

#### 3.4.1. Heterogeneity

This systematic review addressed heterogeneity using a random-effects model because this method is free of major methodological challenges. In terms of sensitivity, the forest plot and subgroup analysis demonstrate substantial heterogeneity between studies (I^2^ = 72%, *p* = 0.000 < 0.05). In relation to specificity, extreme heterogeneity was detected (I^2^ = 93%, *p* = 0.000 < 0.05). For the Chi-square test, *p* = 0.000, which confirms the alternative hypothesis and hence heterogeneity between studies.

#### 3.4.2. Publication Bias

**Figure 6:**
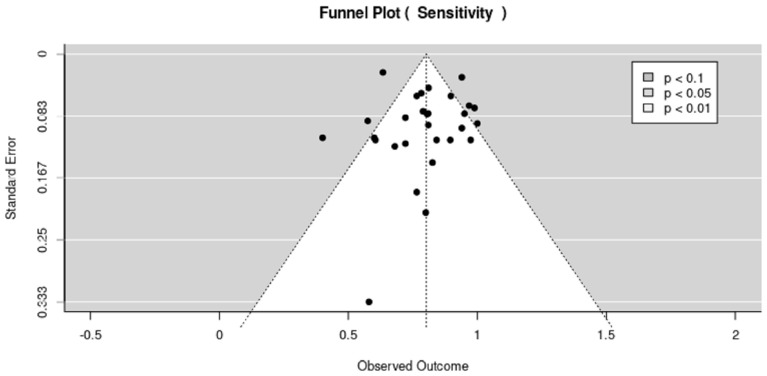
Funnel plot sensitivity for all modalities.

**Figure 7:**
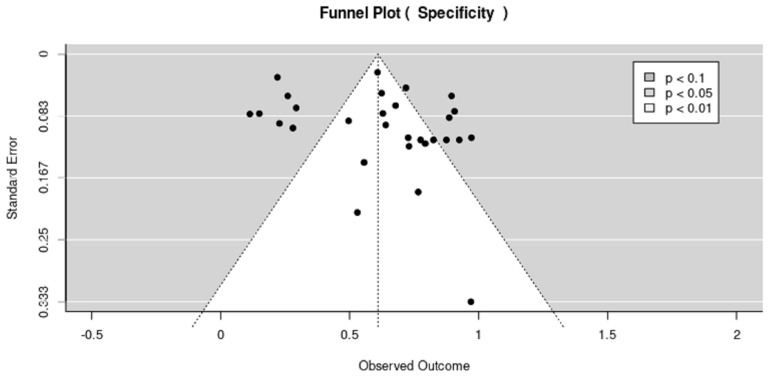
Funnel plot specificity for all modalities.

The funnel plot is based on the estimation of effect size that increases with the sample size of each study. The effect size increases as the sample size increases. The results in Figure 6 and Figure 7 show the publication bias represented in the funnel plots for sensitivity and specificity, respectively. The dot on the scatter plots is for the individual studies included in the systematic review, where each dot represents each study. The results in Figure 6 and Figure 7 show that the funnel plot is clearly asymmetric, meaning that there is publication bias for both sensitivity and specificity.

## 4. Discussion

### 4.1. Main Findings of the Study in the Context of the Reported Literature

In our systematic review, we have assessed the diagnostic test accuracy of different ultrasound scanning technologies (shear-wave elastography, contrast enhanced, micro ultrasound) and grayscale ultrasound technology in the detection of prostate cancer. Published research evaluating the sensitivity and specificity of Micro-US, mpUS, grayscale, elastography and CEUS modalities for the diagnosis of prostate cancer in symptomatic patients revealed that the pooled sensitivity and specificity for all studies combined was 0.80 (95% CI 0.75–0.86) and 0.61 (95% CI 0.51–0.71), respectively. Positive predictive values varied from 0.35 to 0.97, while the negative predictive values ranged between 0.31 and 0.97. The clinical relevance of these tumors is debatable, even though sensitivity of 0.80 (95% CI 0.70–0.84) predicts 20% false-negative individuals. Overall, the quality of the included studies was considered low risk.

It has been argued that the current industry-standard imaging method for prostate biopsies is conventional transrectal grayscale ultrasound [55,56]. Grayscale is employed in brachytherapy, systematic biopsies, volumetry and seed-placement guidance [56,57]. According to the studies collected for the current systematic review, the sensitivity of GSU for detecting prostate cancer varied between 58 and 81% and the specificity ranged between 11 and 93%. In addition, the current study found that the pooled sensitivity and specificity for grayscale were 0.66 (95% CI 0.54–0.73) and 0.56 (95% CI 0.21–0.90), respectively. On the other hand, according to a systematic review conducted by Postema et al. [14], the sensitivity of grayscale for detecting potential tumors can reach 60%. In addition, their review of the literature revealed that the sensitivity and specificity of grayscale ranged from 8 to 88% and from 42.5 to 99%, respectively.

CEUS is regarded as an ultrasound imaging test, which advances the identification of malignant tumors significantly [58]. According to the current systematic review, the sensitivity of CEUS ranged between 40 and 84%, while the specificity ranged between 64 and 97%. Moreover, the current study revealed that the pooled sensitivity and specificity for CEUS were 0.73 (95% CI 0.58–0.88) and 0.78 (95% CI 0.67–0.88), respectively. Similarly, another systematic review and meta-analysis study of sixteen studies involving 2624 patients was reported that the pooled sensitivity and specificity of CEUS imaging for PCa identification were 70% and 74%, respectively [59].

Moreover, SWE is considered a cutting-edge method for determining stiffness by estimating the speed at which a shear wave moves through the tissues [60,61]. According to the current systematic review, the sensitivity of SWE ranged between 58 and 97%, while the specificity ranged between 56 and 97%. In our study, we found that the pooled sensitivity and specificity for SWE were 0.82 (95% CI 0.75–0.90) and 0.76 (95% CI 0.65–0.88), respectively. A systematic and meta-analysis review of the diagnostic performance of SWE in the detection of prostate cancer revealed a pooled sensitivity and specificity of 0.83 (95% CI, 0.66–0.92) and 0.85 (95% CI, 0.78–0.90), respectively [18]. In addition, Zhang et al. [62] observed that the pooled sensitivity and specificity of seven investigations involving 508 individuals were 0.72 (95% CI, 0.70–0.74) and 0.76 (95% CI, 0.74- 0.78), respectively. Another meta-analysis by Teng et al. [63] evaluated the performance of strain elastography-targeted biopsy and revealed a pooled sensitivity and specificity of 0.62 (95% CI, 0.55–0.68) and 0.79 (95% CI, 0.74–0.84), respectively.

Furthermore, micro-ultrasound modality is considered a novel imaging technique that uses high frequencies. From the 11 studies reporting findings on the use of Micro-US as a detection modality for prostate cancer, the current study reported that the sensitivity and specificity ranged from 0.68 to 1 and from 0.22 to 0.92, respectively. The pooled sensitivity and specificity for the Micro-US were 0.85 (95% CI 0.76–0.94) and 0.43 (95% CI 0.28–0.59), respectively. A recent meta-analysis of seven studies with 769 patients found that micro-ultrasound has sensitivity and specificity values of 0.91 and 0.49, respectively [64]. Their study results reported a pooled sensitivity of 0.91 (95% confidence interval (CI) 0.79–0.97) and a pooled specificity of 0.49 (95% CI 0.30–0.69). They concluded that the capacity to identify the presence of prostate cancer using Micro-US was robust, yet the likelihood of misdiagnosis was significant.

Zhang et al.’s [30] study on 78 patients with an elevated or increasing PSA level (>4.0 ng/mL) found that TRUS, SWE and CEUS techniques could not reliably diagnose PCa on their own. The current meta-analysis showed a pooled sensitivity and specificity for the mpUS of 0.87 (95% CI 0.71–1.03) and 0.68 (95% CI 0.54–0.81), respectively. To our knowledge, only two studies have been conducted on mpUS for the detection of prostate cancer. Zhang et al. [30] reported the sensitivity, specificity, PPV and NPV as 97%, 78%, 90% and 97% respectively. Their study showed that multiparametric TRUS performed better in terms of diagnosis. The sensitivity and NPV were as high as 97.4% and 96.9%, respectively. Moreover, the accuracy was 87.2%, and the area under the receiver operating characteristic curve was higher than that for MRI at 0.874 and 0.043, when the TRUS, SWE or CEUS involved the favored malignancy being diagnosed as PCa. MRI had higher sensitivity and NPV than multiparametric TRUS, but poorer specificity and positive predictive value. Additionally, although additional evidence is required to support this theory, patients using multiparametric TRUS may avoid needless biopsies and experience lower medical expenses and consequences. However, Zhang et al.’s [30] study has few limitations, for instance, the sample size was considered small. Furthermore, only 12 PCa patients overall had radical prostatectomy together with surgical pathologic evaluations. In using a TRUS-guided biopsy to diagnose the other 26 instances, a sample error could not be ruled out.

Another study conducted by Zhang et al. (2022) assessed the diagnostic efficacy of mpUS and mpMRI-TRUS fusion for csPCa on 140 patients with PSA > 4 ng/mL. Their study reported the sensitivity, specificity, PPV and NPV as 84%, 63%, 71% and 78%, respectively [42]. However, their study has several limitations. For instance, they used the puncture results as the gold standard for the 140 patients who were not part of the 20 patients who received radical prostatectomy. First-time biopsy procedures were performed on all patients; therefore, radical prostatectomy was not performed if the patient’s biopsy result was negative, which may have resulted in missed diagnoses of low-grade and some advanced PCa. To prevent selection bias, they chose to perform pre-biopsy MRI on patients who did not have any contraindications other than biopsy based on MRI risk assessment. Additionally, they integrated mpUS with mpMRI TRUS fusion imaging. The fusion region’s imaging characteristics were subsequently further defined, which was thought to be useful for accurately localizing PCa and carrying out the subsequent puncture procedure.

### 4.2. Limitations of the Review

Although this systematic review was carried out strictly, precisely and methodically, which was advantageous for this study, it contains several drawbacks. First, most of the included studies were non-randomized and single-institutional studies. To confirm our findings, additional randomized multicenter investigations are required. Another limitation is that all included studies only performed the reference test on individuals who had elevated PSA levels or abnormal prostate exams, which may lead to verification bias. Therefore, the true sensitivity of PSA in symptomatic patients is unknown and probably lower than stated. Combining the modalities may increase the number of tumors that are detected while improving specificity due to the increased evaluation of concerning lesion features. However, there are limited data on the performance of mpUS in the detection of prostate cancer. The current review identified only two studies that assessed the performance of the mpUS in the diagnosis of prostate cancer, which was not enough data to compare with the other modalities.

### 4.3. Clinical Implications of the Review

Based on the findings of this study, we recommend the following:

- Transrectal ultrasonography (TRUS) is a frequently employed method for prostate imaging and biopsy guiding. It is known to allow the prostate gland to be more visible and assists in the detection of any abnormal areas. Thus, we recommend that clinicians combine magnetic resonance imaging and TRUS to accurately identify prostate cancers. Real-time ultrasound and previously acquired MRI images can be combined in this way to improve the visibility of any questionable lesions and direct biopsy needles to the right places.
- Micro ultrasound, a more recent imaging technique, provides better prostate visibility and resolution than traditional ultrasound. Thus, we recommend that clinicians use micro ultrasound to accurately detect prostate cancer as it increases the accuracy of biopsies and decreases unnecessary procedures. In addition, it is thought to improve the detection and localization of any questionable lesions within the prostate.
- Multi-parametric ultrasound can be applied longitudinally to track disease development and evaluate treatment effectiveness. Clinicians can assess modifications to tumor size, vascularity and tissue features over time by comparing serial mpUS scans. These data can aid in assessing the efficacy of therapy, spotting recurrent illness and directing future management choices.
- Compared to other imaging modalities such as MRI, mpUS can be carried out by utilizing either transrectal or transperineal techniques, both of which are minimally invasive. This makes mpUS a practical and well-tolerated choice for routine testing and monitoring in prostate cancer patients.
- Targeted biopsies of questionable spots found on imaging can be guided using mpUS. mpUS can precisely identify and locate concerning lesions by combining imaging modalities such as B-mode, contrast-enhanced ultrasound and elastography. This increases the chance of finding prostate cancer and decreases the number of unnecessary biopsies by enabling more-accurate and focused sampling during biopsies.
- mpUS can also assist in categorizing prostate cancer risk. Using a variety of measurements, including tumor size, vascularity and tissue stiffness, mpUS can determine the cancer’s aggressiveness and stage. Clinicians can use this risk stratification to guide their planning and decision-making for patient care, assisting them in selecting the best course of action.

### 4.4. Research Implications of the Review

Based on the results obtained from this review, we recommend the following:

- There is still a lack of studies on the performance of mpUS in the detection of prostate cancer. Thus, future research should ideally focus on the diagnostic accuracy of mpUS. In addition, further studies are required on the diagnostic accuracy of Micro-US, mpUS, grayscale, elastography and CEUS modalities in asymptomatic men for the early detection of prostate cancer, although this would require large populations and may be very expensive.
- We recommend comparative evaluation of various ultrasound modalities. For instance, there is still a lack of studies that compare the performance and diagnostic efficacy of different ultrasound technologies used to find prostate cancer. Transrectal ultrasound (TRUS) and transperineal ultrasound (TPUS) can be compared, and the efficacy of fusion imaging—which combines traditional ultrasound with an assessment of the prospective advantages of new technologies such as micro ultrasound—can also be evaluated.
- We recommend future research on the viability and efficacy of more recent ultrasound methods for the detection of prostate cancer, such as micro ultrasonography, contrast-enhanced ultrasound (CEUS) and multiparametric ultrasound, and to investigate how such methods assist in improving the sensitivity, specificity and location of suspected lesions inside the prostate gland.
- We recommend future validation studies to evaluate the effectiveness of ultrasound technologies in a range of patient populations, including those with various risk profiles or clinical traits. This can assist in determining the generalizability and usability of ultrasound techniques for the detection of prostate cancer in different contexts.
- We recommend future longitudinal research on the long-term results and influence on patient care, for instance, the long-term effects and impact on patient management of the application of various ultrasound technologies for the identification of prostate cancer. To establish the therapeutic relevance and consequences of these technologies, future research should consider elements including biopsy accuracy, treatment decision-making, surveillance techniques and patient outcomes.

## 5. Conclusions

This systematic review and meta-analysis show that some studies proved that advanced ultrasound modalities such as mpUS, Micro-US, shear-wave elastography, and contrast-enhanced and micro ultrasound are promising methods for the detection of prostate cancer. These techniques serve to address the ever-increasing burden on MRI and its drawbacks, including lack of access, inconsistency in MRI acquisition and interpretation, and real-time imaging for precise targeted biopsy, while also adding vital information to the diagnostic route for prostate cancer.

## Figures and Tables

**Table 1:** Inclusion and Exclusion criteria.

	Inclusion	Exclusion
Settings	All Countries	None
Participants	Male patients (all ages) with a suspicion of prostate cancer, based on an elevated serum PSA concentration or abnormal digital rectal examination	Females
Modality	Studies that used the following devices: Multiparametric ultrasound Micro ultrasound Grayscale Elastography Contrast-enhanced ultrasound	Studies that did not use the following devices: Multiparametric ultrasound Micro ultrasound Grayscale Elastography Contrast-enhanced ultrasound
Outcomes	Studies that report sensitivity, specificity, positive predictive value and negative predictive value	Studies that do not report sensitivity, specificity, positive predictive value or negative predictive value
Study Type	In vivo studies Prospective and retrospective studies Randomized Clinical trial Non-randomized	In vitro studies Review articles Systematic review
Publication Type	Journal articles	Conference abstract, study protocol, report, dissertation, books and non-professional journal
Publication Year	Publication date 2018 and after	Publication date before 2018
Language	English	All other languages

**Table 2:** Results of Meta-Analysis.

Modality	Authors (Year)	Number of Cases	Sensitivity (%)	Specificity (%)	PPV	NPV	Accuracy
**Micro-US**	Chessa et al. (2021) [32]	68	68	73	93	31	69
	Cornud et al. (2020) [33]	118	100	23	75	100	77
	Fasulo et al. (2022) [34]	140	72	89	83	81	
	Klotz et al. (2020) [35]	1040	94	22	44	85	50
	Lopci et al. (2021) [36]	25	80	53	35	89	61
	Lorusso et al. (2022) [37]	32	77	77	64	86	77
	Lughezzaniet al. (2019) [39]	104	94	28	40	90	49
	Lughezzani et al. (2021) [38]	320	90	26	41	82	49
	Pavlovich et al. (2021) [31]	1676	19	92	63	61	
	Socarras et al. (2020) [41]	194	99	29	62	96	
	Wiemer et al. (2021) [40]	159	95	15	52	75	54
**mpUS**	Zhang et al. (2018) [30]	78	97	78	80	97	87
	Zhang et al. (2022) [42]	160	84	64	72	79	
**Grayscale**	Lee et al. (2018) [45]	157	81	11	47	38	
	Liu et al. (2020) [44]	82	60	73	71	63	67
	Yoo and Lee (2022) [43]	127	58	50	33	73	52
	Zhang et al. (2018) [30]	78	61	93	89	71	77
**Shear wave elastography**	Fu et al. (2019) [51]	172	79	91	71	94	
	Shah et al. (2019) [50]	50	83	56	61	79	68
	Shoji et al. (2018) [48]	12	58	97	86	87	
	Su et al. (2018) [49]	320	77	90	83	85	
	Wei et al. (2018) [46]	212	97	68			96
	Xiang et al. (2019) [47]	367	79	62	47	87	67
	Zhang et al. (2018) [30]	78	90	83	83	89	86
**CEUS**	Drudi et al. (2019) [53]	82	40	97	94	55	63
	Liu et al. (2022) [52]	490	81	72	97	92	74
	Pang et al. (2022) [9]	72	72	79	84	66	75
	Postema et al. (2020) [54]	113	81	64			
	Zhang et al. (2018) [30]	78	84	88	87	85	86

**Table 3:** Characteristics of Selected Studies for Micro-US.

Author (Year)	Study Design	Population	Methodology	Device	Biopsies/Radical Prostatectomy	Outcome Measures	Conclusions
**Chessa et al. (2021) [32]**	Prospective database	68 patients with biopsy-proven PCa.	Patients received mpMRI, which resulted in the discovery of an index lesion with a PIRADS-v2 score of at least 3. A fusion biopsy was performed. Micro-ultrasound images were taken of all males who had prostate cancer at the level of the index lesion as determined by biopsy.	ExactVu^TM^ and mpMRI	Fusion biopsy was imaged by ExactVuTM	Sensitivity, specificity, PPV and NPV.	A high resolution of the prostatic peripheral zone is provided by ExactVuTM, which may advance the triage tool’s ability to identify csPCa.
**Cornud et al. (2020) [33]**	Retrospective single-center	118 patients with a rising PSA level.	Micro-US-guided biopsy performed on patients with MRI guidance. All of the lesions that could be seen on the Micro-US were targeted without the use of image fusion, which was performed for the lesions that could be seen on the MRI and/or the micro ultrasound.	Micro-US and bp-MRI	MRI and TRUS-guided biopsies	Sensitivity and specificity.	The use of Micro-US as a complementary examination to bp-MRI may provide some potential in its ability to localize targets.
**Fasulo et al. (2022) [34]**	Prospective	140 patients with biopsy-proven prostate cancer.	A side-free endorectal probe and a 29 MHz ExactVuTM Micro-US device were used for Micro-US imaging on all patients the day before RARP.	Micro-US	Radical prostatectomy	Sensitivity, specificity, negative predictive value and positive predictive value. AUC.	Micro-ultrasound could effectively predict EPE in patients scheduled for RARP based on the final pathology report.
**Klotz et al. (2020) [35]**	Multi-center prospective registry	1040 patients were diagnosed with PCa based on abnormal digital rectal examination and/or increased PSA.	Biopsies were collected from micro-ultrasound and mpMRI targets. Systematic biopsy was taken up to 14 cores.	Micro ultrasound and mpMRI	Micro ultrasound and mpMRI	Sensitivity, specificity, negative predictive value (NPV) and positive predictive value (PPV).	In comparison to mpMRI, micro ultrasound demonstrated a similar or greater sensitivity and similar specificity for csPCs. For targeted biopsy and prostate screening, micro ultrasound provides an inexpensive, one-session selection.
**Lopci et al. (2021) [36]**	Pilot prospective single-institutional clinical trial	25 patients with suspicion of prostate cancer.	Patients were given 68 Ga-PSMA PET/TRUS fusion biopsy assignments, and their results were compared to PRI-MUS system grading.	68 Ga-PSMA PET/CT, Micro-US, TRUS biopsy	Comparison of PRI-M with Ga-PSMA PET/TRUS fusion biopsy	Sensitivity, specificity, negative predictive value (NPV) and positive predictive value (PPV).	The diagnostic performance of 68Ga-PSMA PET/CT is better than PRI-MUS protocol.
**Lorusso et al. (2022) [37]**	Retrospective	32 patients with biopsy-proven PCs.	Patients diagnosed with prostate cancer using micro-ultrasound imaging and scheduled for radical prostatectomy.	Micro-US and mpMRI	Radical prostatectomy	Sensitivity, specificity, negative and positive predictive values, and accuracy.	In diagnosing prostate cancer index lesions, micro ultrasound showed high reliability, comparable to mpMRI in terms of performance.
**Lughezzani et al. (2019) [39]**	Prospective single-institutional clinical trial	104 patients with a clinical suspicion of prostate cancer who were examined consecutively.	All patients had micro-ultrasound-targeted biopsies conducted by urologists who were unaware of the results of the mpMRI scans. Substantially, 12-core systematic and MRI/US-fusion-targeted biopsy were performed.	Micro-US and MRI/US	Micro-US-targeted biopsiesMRI/US fusion targeted	Sensitivity, specificity, positive predictive value (PPV) and negative predictive value (NPV).	Micro ultrasound can provide more details about the absence or presence of csPCa in patients who have clinical suspicion of prostate cancer.
**Lughezzani et al. (2021) [38]**	Prospective cohort	320 patients with a suspicion of PC based on high PSA test.	Patients had micro-ultrasound scan prior to biopsy utilizing ExactVu system.	MRI and Micro-US	MRI and Micro-US	Sensitivity, specificity, negative predictive value (NPV) and positive predictive value (PPV).	For directed prostate biopsies, micro ultrasound is a capable imaging scanning technique.
**Pavlovich et al. (2021) [31]**	Prospective randomized clinical trial	1676 candidates received prostate biopsy with unknown PCa.	One of two biopsy techniques—conventional ultrasonography or micro ultrasound—was assigned randomly to each patient.	Conventional ultrasound and micro ultrasound	Conventional ultrasound and micro ultrasound	Per-patient detection of csPCa.	Micro-US was not clearly superior to conventional ultrasound in detecting csPC during biopsy.
**Socarras et al. (2020) [41]**	Retrospective	194 patients with suspicion of PCa.	Transperineal prostate biopsies technique utilizing ultrasound fusion targeted biopsy and real-time targeted Micro-US were performed on all patients.	Micro-ultrasound-guided biopsy and multiparametric MRI	Transperineal biopsies	Sensitivity, specificity, PPV and NPV.	High diagnostic accuracy for csPCa and PCa, preventing infectious complications associated with biopsy.
**Wiemer et al. (2021) [40]**	Prospective cohort	159 patients with a clinical suspicion of PCa.	Patients with clinical suspicion of prostate cancer had TRUS biopsy by micro-ultrasound (ExactVu) system. Prior to prostate biopsy, all patients underwent mpMRI.	NTB, MRI-TB, Micro-US-TB, NTB + MRI-TB, NTB + Micro-US-TB, Micro-US-TB + MRI-TB	Systematic biopsy and targeted cores	Sensitivity, specificity, negative predictive value (NPV) and positive predictive value (PPV).	Micro-US has advantages over mpMRI-targeted biopsies. It is feasible to replace conventional ultrasonography and eliminate routine systematic biopsies in a unique biopsy strategy that uses Micro-US and mpMRI only for targeted biopsies.

## Data Availability

The data presented in this study are available within this article.

## Supplementary Materials

The following supporting information can be downloaded at: https://www.mdpi.com/article/10.3390/cancers15164105/s1, Figure S1: Pooled sensitivity for Micro-US [31,32,33,34,35,36,37,38,39,40,41], Figure S2: Pooled specificity for Micro-US [31,32,33,34,35,36,37,38,39,40,41], Figure S3: Pooled sensitivity for mpUS [30,42], Figure S4: Pooled specificity for mpUS [30,42], Figure S5: Pooled sensitivity for grayscale ultrasound [30,43,44,45], Figure S6: Pooled specificity for grayscale ultrasound [30,43,44,45], Figure S7: Pooled sensitivity for SWE [30,47,48,49,50,51], Figure S8: Pooled specificity for SWE [30,47,48,49,50,51], Figure S9: Pooled sensitivity for CEUS [9,30,52,53,54], Figure S10: Pooled specificity for CEUS [9,30,52,53,54]; Table S1: Characteristics of Selected Studies for mpUS, Table S2: Characteristics of Selected Studies for Grayscale, Table S3: Characteristics of Selected Studies for SWE, Table S4: Characteristics of Selected Studies for CEUS.
