# Supplementary material for: Comparative Assessment of Different Ultrasound Technologies in the Detection of Prostate Cancer: A Systematic Review and Meta-Analysis
Source: Cancers (Basel). 2023 Aug 15;15(16):4105. doi: 10.3390/cancers15164105 (PMC10452802; doi:10.3390/cancers15164105)
Supplement: Supplementary file 1 [file cancers-15-04105-s001.zip › supplementary tables of Study characterstics.pdf]

Table S1: Characteristics of Selected Studies for mp-US

Author (Year)	Study design	Number of Patients	Methodology	Device	Biopsies/radical prostatectomy	Outcome Measures	Conclusion
**Zhang et al. (2018) [30]**	Prospective	78 patients with increasing PSA.	38 PCa patients and 40 benign patients. Twelve of the 38 PCa patients had radical prostatectomy.	Multiparametric transrectal ultrasound (TRUS), including grayscale ultrasound, colour Doppler ultrasound, shear wave elastography, and contrast-enhanced ultra-sound.	Systematic US-guided TRUS biopsies and radical prostatectomy were performed	Sensitivity, specificity, positive predictive value, and negative predictive value.	In the diagnosis of localised prostate cancer (≤2 cm), the diagnostic performance of multiparametric MRI and multiparametric TRUS are equivalent.
**Zhang et al. (2022) [42]**	Retrospective	A month prior to biopsies, mpMRI was performed on 160 patients with PSA>4 ng/mL.	Patients who had never had a biopsy received mpMRI-TRUS fusion imaging along with targeted and systematic mpUS-guided biopsies.	Multiparametric ultrasonography (mpUS) and multiparametric magnetic resonance imaging/transrectal ultrasound (mpMRI-TRUS).	mpUS-guided targeted biopsies (TB) and systematic biopsies (SB) and radical prostatectomy	Sensitivity, specificity, positive predictive value, and negative predictive value.	A targeted biopsy may be successfully employed for diagnosis and risk assessment of csPC because it can increase the rate of csPC detection. For mpMRI-TRUS fusion imaging, the mpUS features of the target regions may provide important diagnostic data.

Table S2: Characteristics of Selected Studies for Grayscale

Author (Year)	Study design	Population	Methodology	Device	Biopsies/radical prostatectomy	Outcome Measures	Conclusion
**Lee et al. (2018) [45]**	Prospective	157 patients with suspected PCa.	Prior to obtaining the 12-core systemic biopsy, 1-core targeted biopsy done for each patient who has up to 2 lesions that appear as hypoechoic lesions.	Grayscale and Hounsfeld units (HU).	14 core biopsies	Sensitivity, specificity, positive predictive value, and negative predictive value. Detection.	To identify PCa and provide more accurate pathologic data for the diagnosis of PCa, hypoechoic lesions may be quantified using grayscale technology and HU.
**Liu et al. (2020) [44]**	Retrospective	82 patients with a suspected PCa.	The patients were divided into two groups, Group 1 (26 males) and Group 2 (42 males), based on who received TRUS biopsy from a prostate contrast-enhanced US examination or who received targeted TRUS guided biopsy.	Grayscale	Systematic 12 core biopsies and 2 targeted biopsies	Sensitivity, specificity, and accuracy.	Before performing a biopsy, a patient with increased PSA may benefit from a CEUS examination of the prostate. This may assist identify males at high risk of prostate cancer, decrease the number of unneeded biopsy, enhance PCa

							detection rates and enhance diagnostic sensitivities and precision.
**Yoo and Lee (2022) [43]**	Prospective	127 patients with a suspected PC.	All patients had 12 targeted biopsy cores for hypoechoic regions and 2 cores target biopsy for 2 more hypoechoic lesions.	Grayscale	14 core targeted biopsies	Sensitivity and specificity.<br><br>Detection	The sensitivity and specificity of a TRUS biopsy might be improved by using a certain range of grayscale values, which could encourage an objective evaluation of lesions.
**Zhang et al. (2018) [30]**	Prospective	78 patients with increasing PSA.	38 PCa patients and 40 benign patients. Twelve of the 38 PCa patients had radical prostatectomy.	Multiparametric transrectal ultrasound (TRUS), including grayscale ultrasound, colour Doppler ultrasound, shear wave elastography, and contrast-enhanced ultra-sound.	12 core biopsies and radical prostatectomy	Sensitivity, specificity, positive predictive value, and negative predictive value.	In the diagnosis of localised prostate cancer (≤2 cm), the diagnostic performance of multiparametric MRI and multiparametric TRUS are equivalent.

Table S3: Characteristics of Selected Studies for SWE

Author (Year)	Study design	Number of Patients	Methodology	Device	Biopsies/radical prostatectomy	Outcome Measures	Conclusion
**Fu et al. (2019) [51]**	Prospective	172 patients with suspected PCa.	Patients received SWE and MRI procedures. In the region that had been prepped for a systematic biopsy, the elastic value was evaluated, and a suspected lesion was found on SWE or B-mode.	Elastography, MRI	Systematic biopsy	Sensitivity, specificity, positive predictive value, and negative predictive value, accuracy.	PCa can be detected using elastography in conjunction with MRI.
**Shah et al. (2019) [50]**	Prospective	50 patients with a clinical suspicion of PC.	Twelve core prostate biopsies were performed on all individuals. Comparing the histology of a core biopsy taken from the affected segment's elastography.	Elastography	12 cores prostate biopsies	Sensitivity, specificity, positive predictive value, and negative predictive value.	Elastography and MRI may both be used and help to identify PCa. With excellent sensitivity and specificity, a cut-off of 90 kPa on SWE may be utilised to distinguish between benign and

							malignant prostate tumours.
**Shoji et al. (2018) [48]**	Prospective	12 patients with PSA levels of 4.0-20.0 ng/ml.	Prior to the biopsies, the patients had 3D SWEs, followed by 12-core systematic biopsies and MRI-TRUS-guided directed biopsy for suspected PCa lesions.	3D Elastography	One biopsy core with a Gleason score of 3+4 or 6 with a maximum cancer core length >4 mm.	Sensitivity, specificity, positive predictive value, and negative predictive value.	The tissue elastic value of cancerous regions was detected as being higher in compression than that of those regions that were undetected. Moreover, PI-RADS and 3D SWE measurements of Young's modulus may help to more accurately identify prostate cancer that is clinically significant.
**Su et al. (2018) [49]**	Prospective	320 men with abnormally raised serum PSA levels (>4 ng/ml).	127 individuals had prostate biopsies done. They conducted TRUS, SWE, total PSA, PSAD, digital rectal examination and free PSA/total PSA ratio (F/T) prior to biopsy.	Elastography	Systematic biopsies with 8-12 cores	Sensitivity, specificity, positive predictive value, and negative predictive value. AUC.	This 11-point grading system, which is rest on shear wave elastography and clinical characteristics, has an excellent prognostic capability for PCa.

**Wei et al. (2018) [46]**	Prospective	212 patients at a clinical risk of PC.	Prior to laparoscopic radical prostatectomy, each patient had quantitative stiffness data collected by an endo-cavitary transrectal transducer.	Elastograp hy	Laparoscopic radical prostatectomy	Sensitivity, specificity	Shear wave ultrasound elastography using transrectal technique successfully identified malignance foci and demonstrated substantial differences between benign and malignant tissue.
**Xiang et al. (2019) [47]**	Retrospectiv e	367 patients at a clinical risk of PCa.	Prior to surgery, free PSA (fPSA) and serum prostate-specific antigen (PSA) were assessed.	MRI + SWE	Trans-perineal prostate biopsy guided by transrectal ultrasound	Sensitivity, specificity, positive predictive value, and negative predictive value.	The use of SWE is crucial for detecting csPCa in prostate glands that have tested false negative by MRI.
**Zhag et al. (2018) [30]**	Prospective	78 patients with increasing PSA.	38 PCa patients and 40 benign patients. Twelve of the 38 PCa patients had radical prostatectomy.	Multipara metric transrectal ultrasound (TRUS), including grayscale ultrasound , colour Doppler ultrasound , shear wave elastograp	Systematic US-guided transrectal prostate biopsies and radical prostatectomy	Sensitivity, specificity, positive predictive value, and negative predictive value.	In the diagnosis of localised prosrtate cancer (≤2 cm), the diagnostic performance of multiparametric MRI and multiparametric TRUS are equivalent.

hy, and contrast-enhanced ultra-sound.

Table S4: Characteristics of Selected Studies for CEUS

Author (Year)	Study design	Number of Patients	Methodology	Device	Biopsies/radical prostatectomy	Outcome Measures	Conclusion
**Drudi et al. (2019) [53]**	Prospective	82 patients with an elevated (>4.0 ng/mL) or increasing PSA.	Patients had mpMRI, prostate biopsy, mpUS, digital rectal examination.	(mpMRI), multiparametric ultrasound (mpUS) and US/MRI	Transperineal biopsy of the prostate included 14 or more cores: 2 target biopsies and 6 random biopsies.	Sensitivity and Specificity.<br><br>Detection.	The TRUS/TRES prostate fusion-guided biopsy in conjunction with bpMRI and mpMRI is a reliable and efficient strategy for identifying PCa.
**Liu et al. (2022) [52]**	Retrospective	490 patients with suspected PCa.	Patients who have PSA levels of 4–10 ng/ml had prostate biopsy	(PI-RADS v2.1) and contrast-enhanced ultrasound (CEUS).	12 core systematic biopsy in addition to 1-2 core targeted biopsy	Sensitivity, specificity, positive predictive value, and negative predictive value.	In patients with "grey zone" PSA levels, PI-RADS v2.1 and CEUS were excellent indicators of csPCa and PCa.

**Pang et al. (2022) [9]**	Prospective	72 patients with PCa.	The diagnostic differences between CEUS, MRI, and CEUS combined MRI for prostate cancer were evaluated using biopsy results as the gold standard.	Contrast-enhanced ultrasound, MRI.	Ultrasonography, MRI, and prostate biopsy.	Sensitivity and Specificity	CEUS or MRI demonstrated pretty high specificity and sensitivity for detecting prostate cancerous tumours. The detection rate of prostate cancer is increased when MRI and CEUS are used together.
**Postema et al. (2020) [54]**	Prospective	113 patients with biopsy proven prostate malignant tumours.	Preoperatively, 2D CEUS imaging was performed on all patients. CEUS recordings led to the generation of CUDI maps.	(2D) contrast-enhanced ultrasound (CEUS) imaging and contrast ultrasound dispersion imaging (CUDI)	Radical prostatectomy	Sensitivity and Specificity	CUDI demonstrated comparable efficacy to 2D CEUS.
**Zhang et al. (2018) [30]**	Prospective	78 patients with increasing PSA.	38 PCa patients and 40 benign patients. Twelve of the 38 PCa patients had radical prostatectomy.	Multiparametric transrectal ultrasound (TRUS).	Systematic US-guided transrectal prostate biopsies were performed	Sensitivity, specificity, positive predictive value, and negative predictive value.	In the diagnosis of localised prosrtate cancer (≤2 cm), the diagnostic performance of multiparametric MRI and multiparametric TRUS are equivalent.
