# Supplementary figures and images for: Comparative Assessment of Different Ultrasound Technologies in the Detection of Prostate Cancer: A Systematic Review and Meta-Analysis
Source: Cancers (Basel). 2023 Aug 15;15(16):4105. doi: 10.3390/cancers15164105 (PMC10452802; doi:10.3390/cancers15164105)

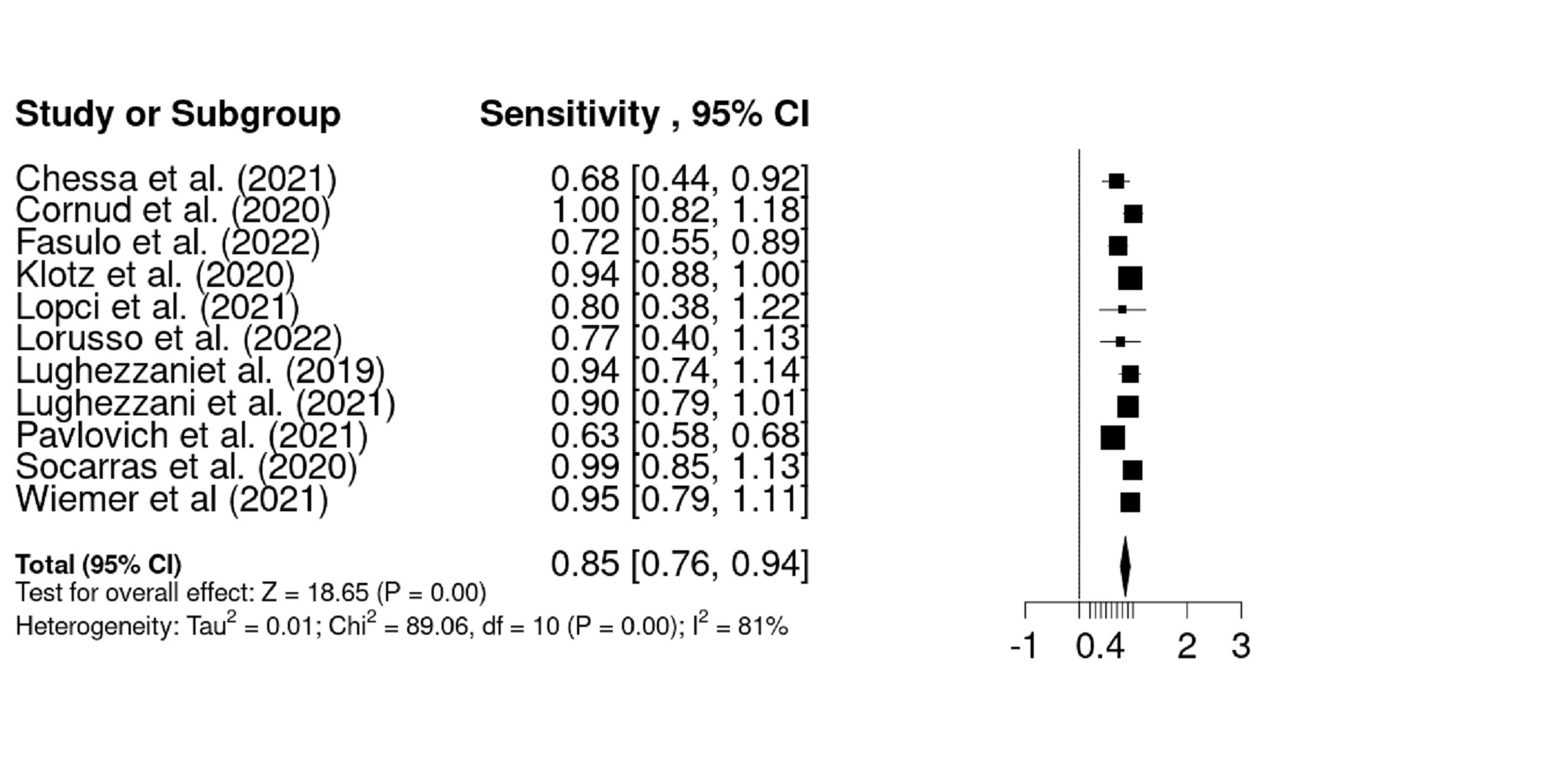

Supplement: Supplementary file 1 [file cancers-15-04105-s001.zip › figure s1 [31-41].jpg]

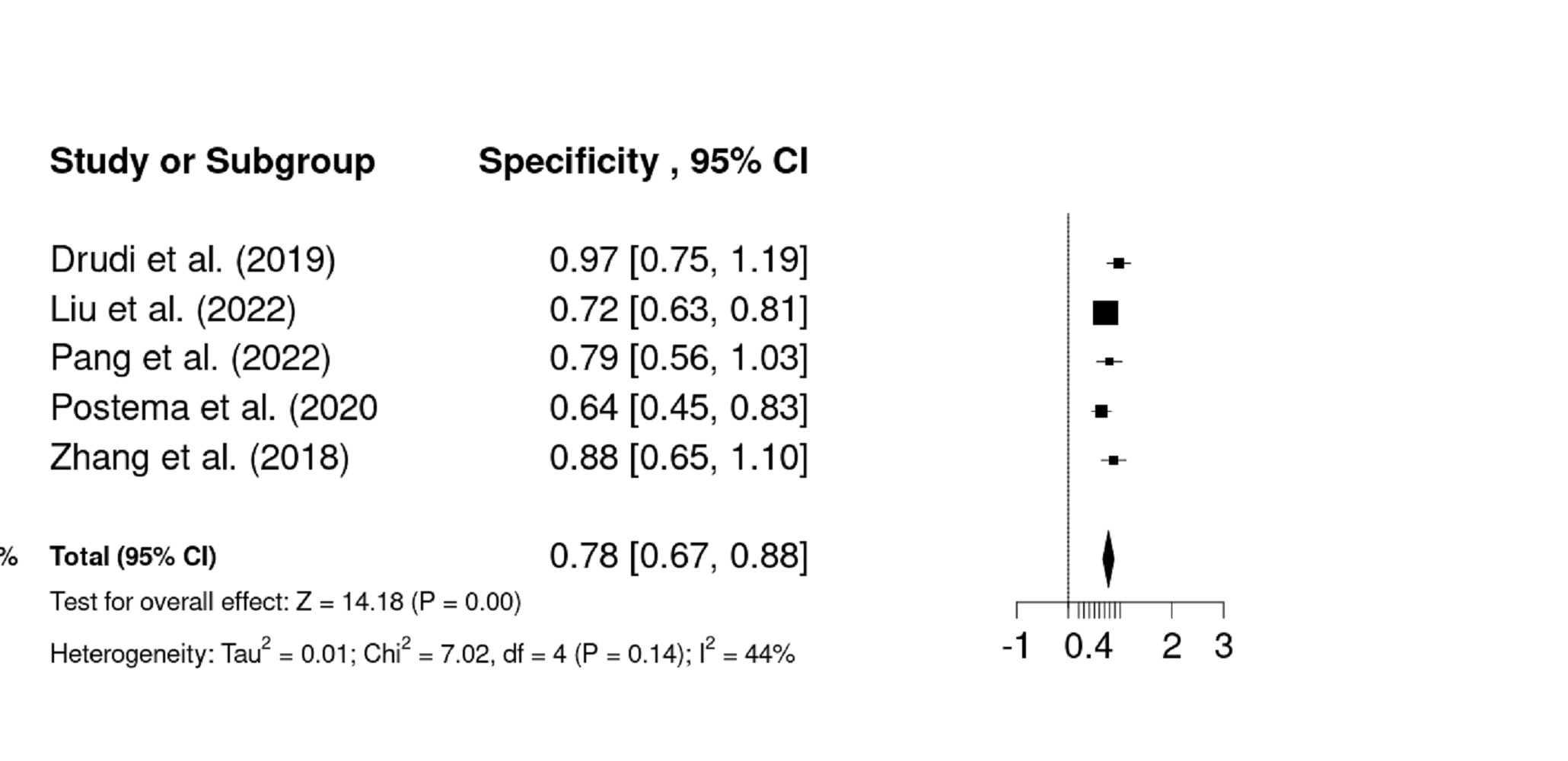

Supplement: Supplementary file 1 [file cancers-15-04105-s001.zip › Figure S10 [9,30,52-54].jpg]

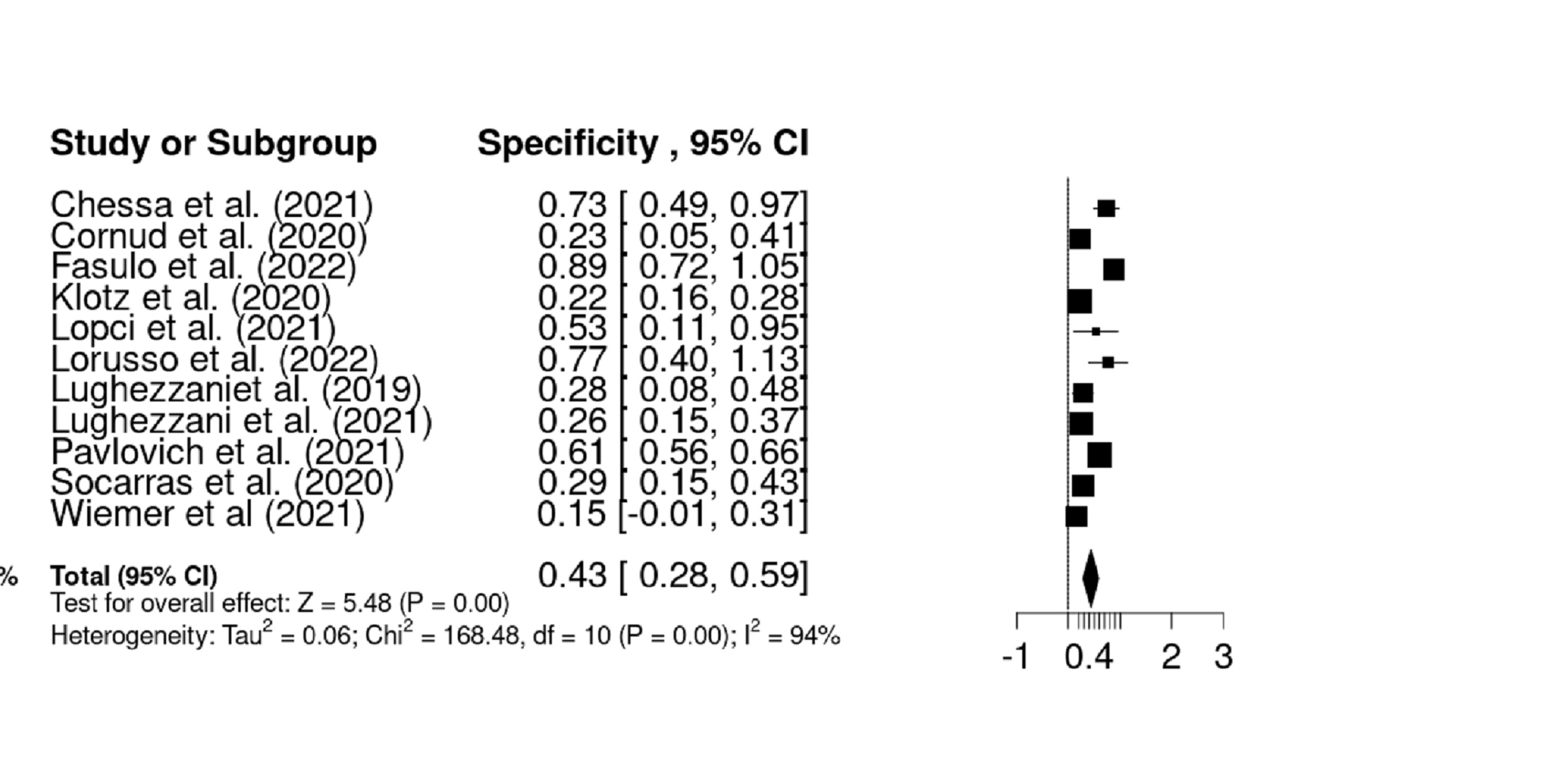

Supplement: Supplementary file 1 [file cancers-15-04105-s001.zip › Figure S2 [31-41].jpg]

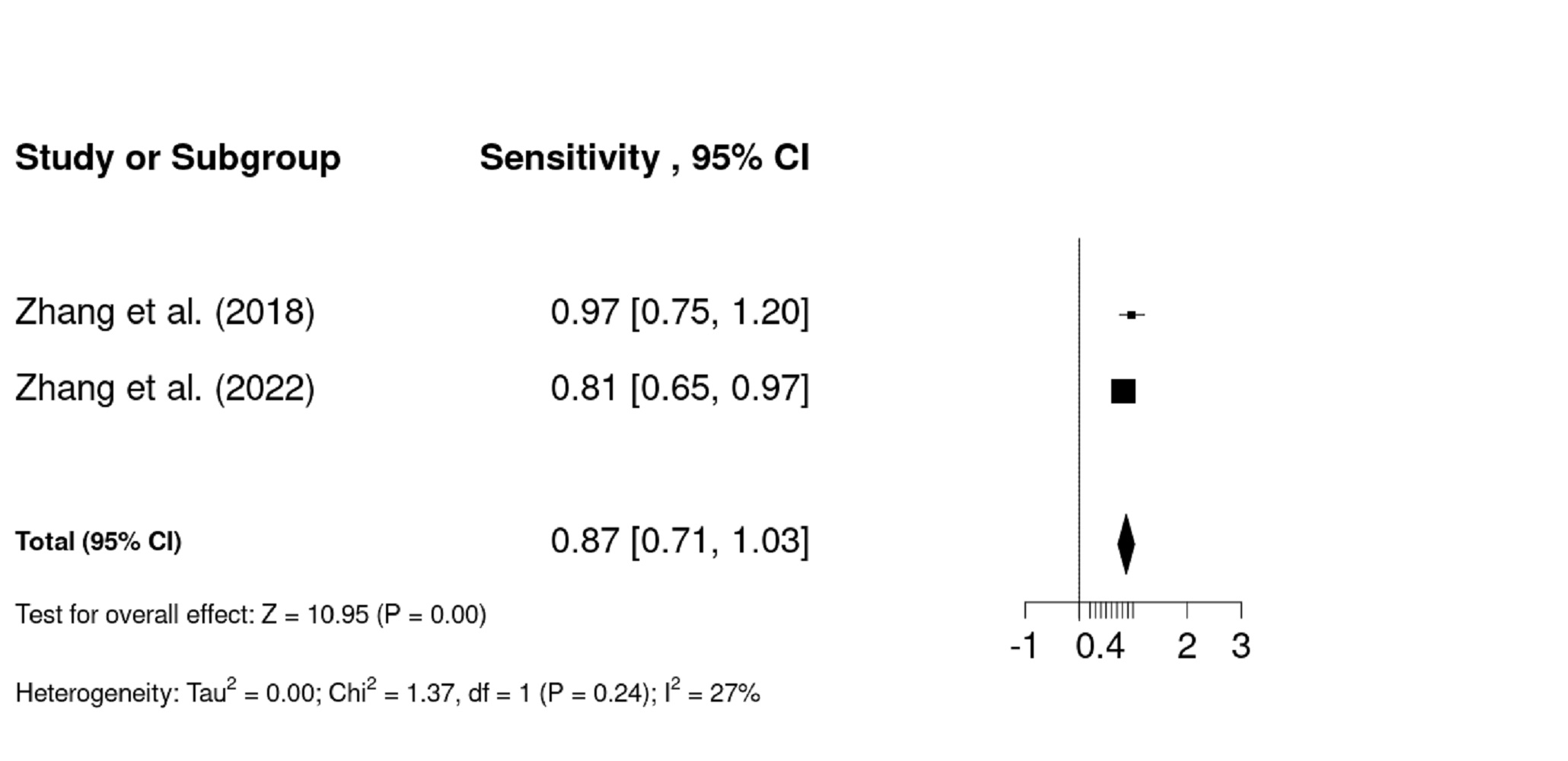

Supplement: Supplementary file 1 [file cancers-15-04105-s001.zip › Figure S3 [30,42].jpg]

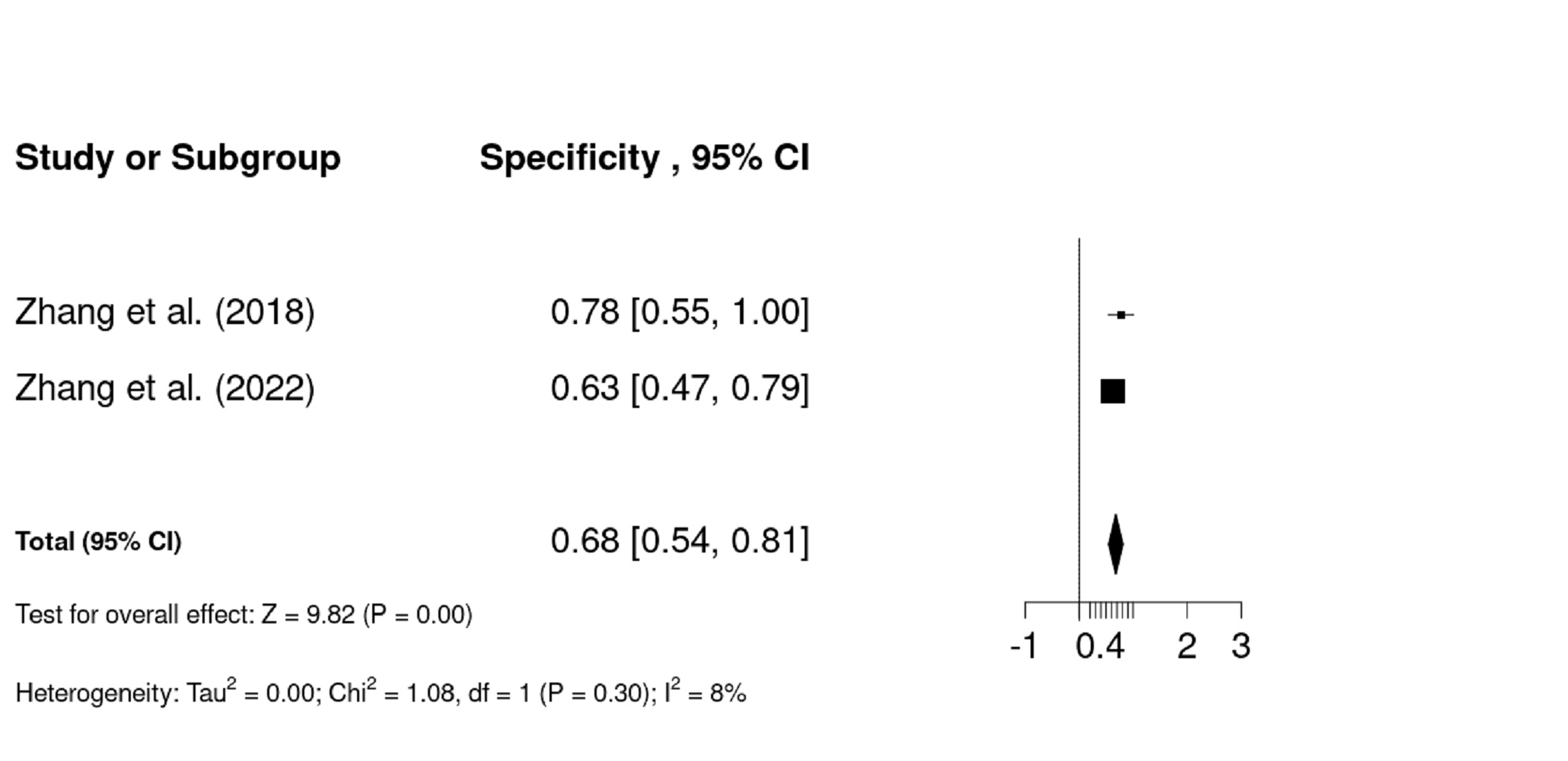

Supplement: Supplementary file 1 [file cancers-15-04105-s001.zip › Figure S4 [30,42].jpg]

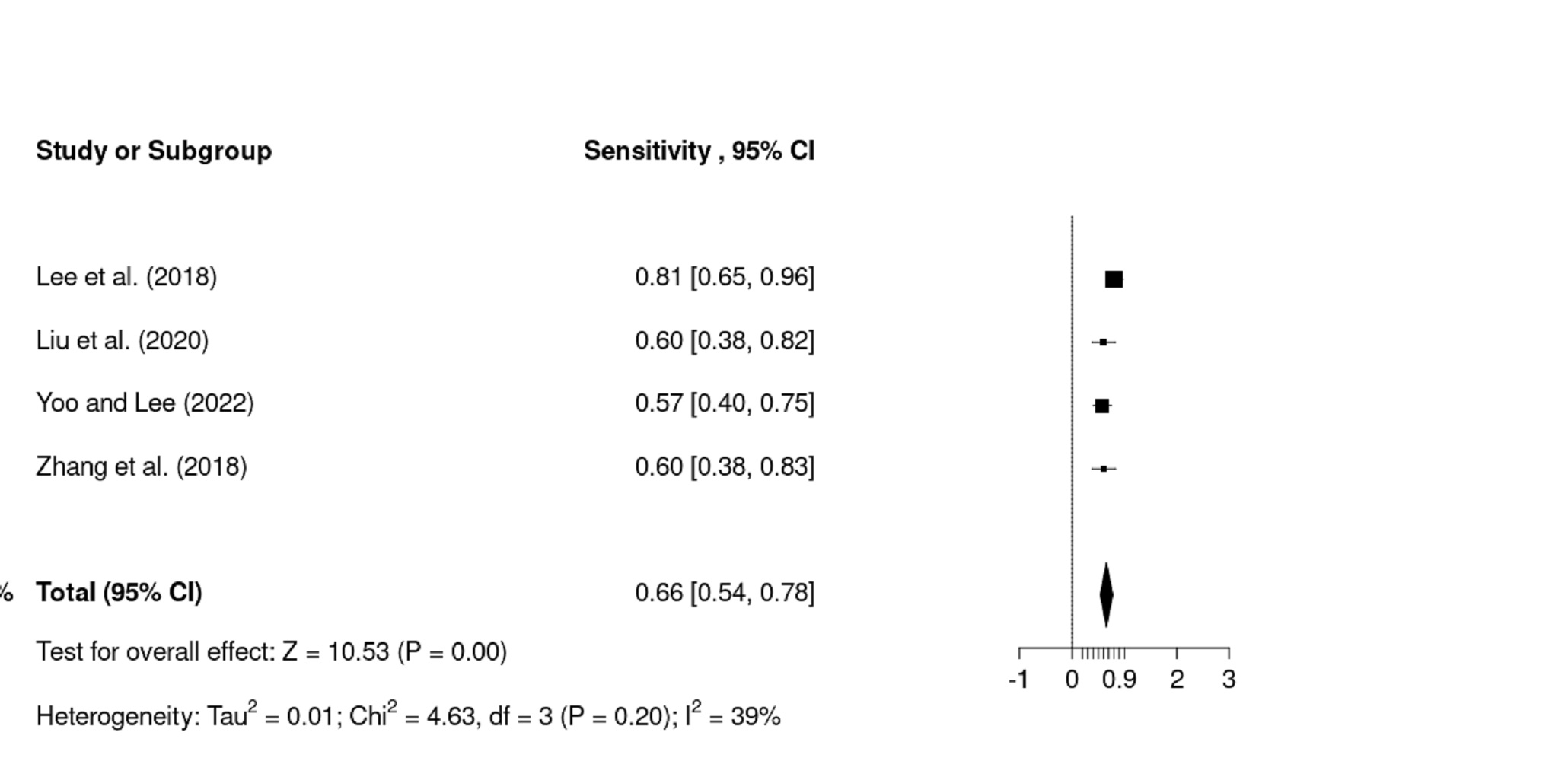

Supplement: Supplementary file 1 [file cancers-15-04105-s001.zip › Figure S5 [30,43-45].jpg]

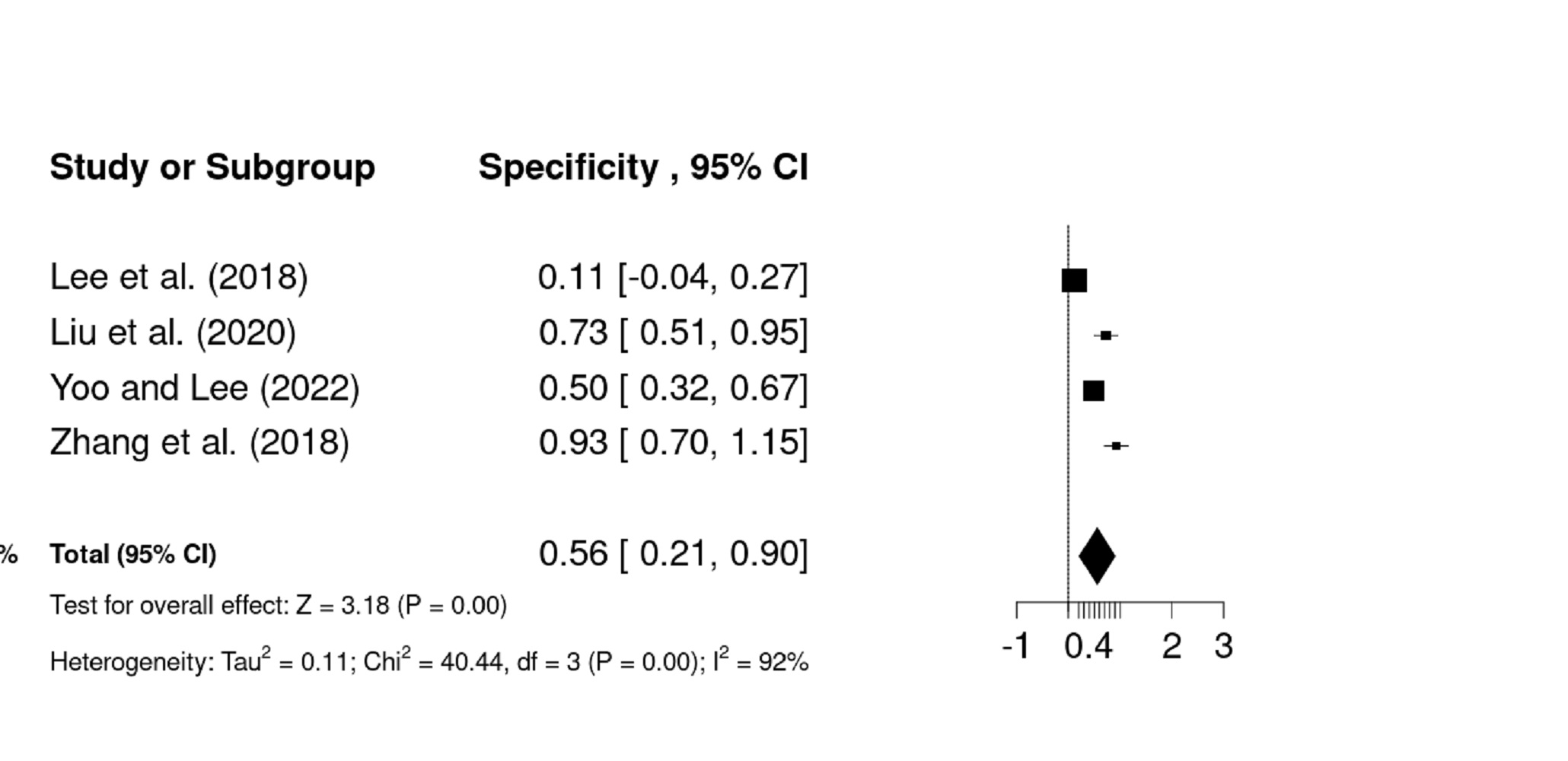

Supplement: Supplementary file 1 [file cancers-15-04105-s001.zip › Figure S6 [30,43-45].jpg]

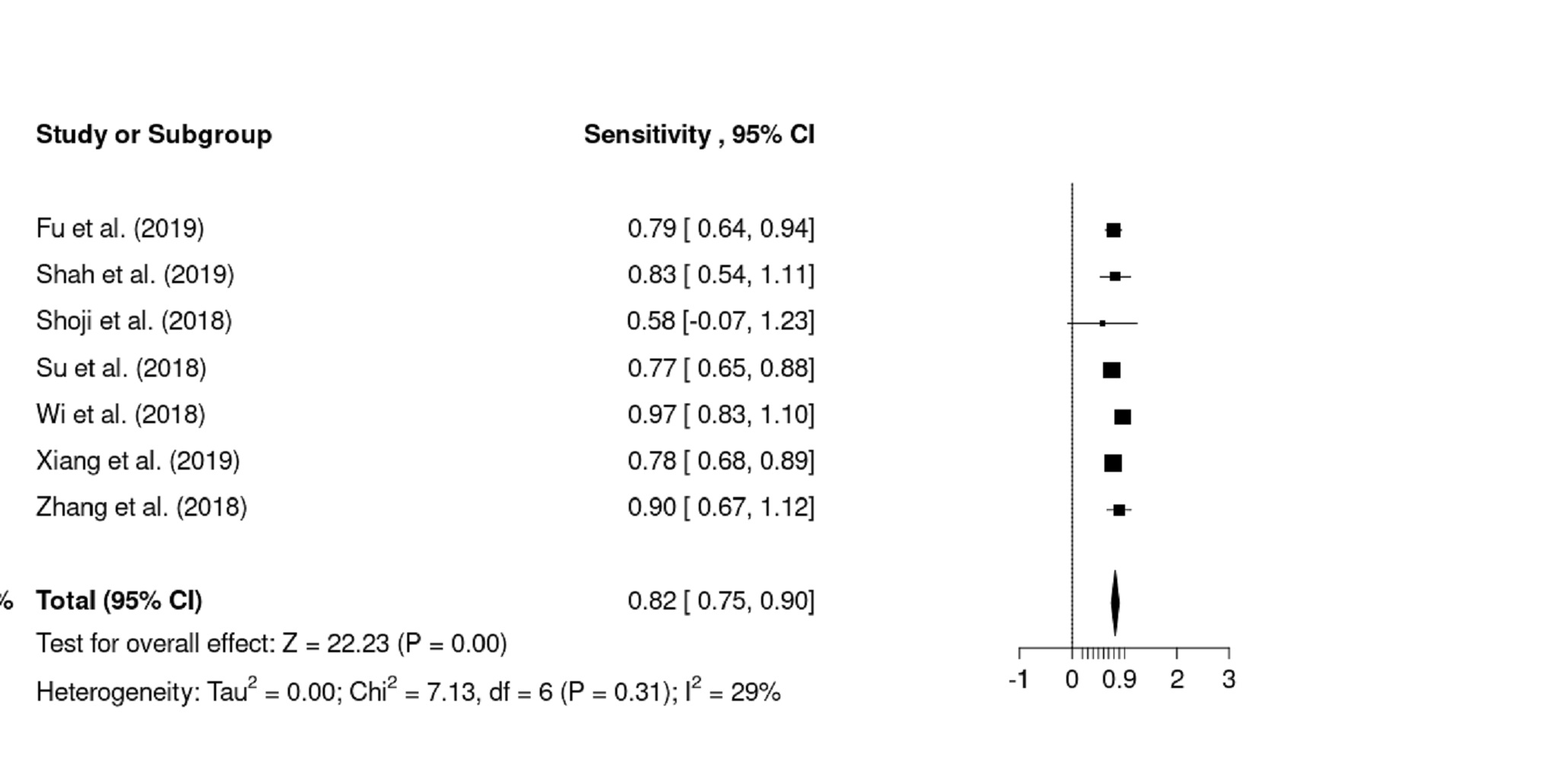

Supplement: Supplementary file 1 [file cancers-15-04105-s001.zip › Figure S7 [30,47-51].jpg]

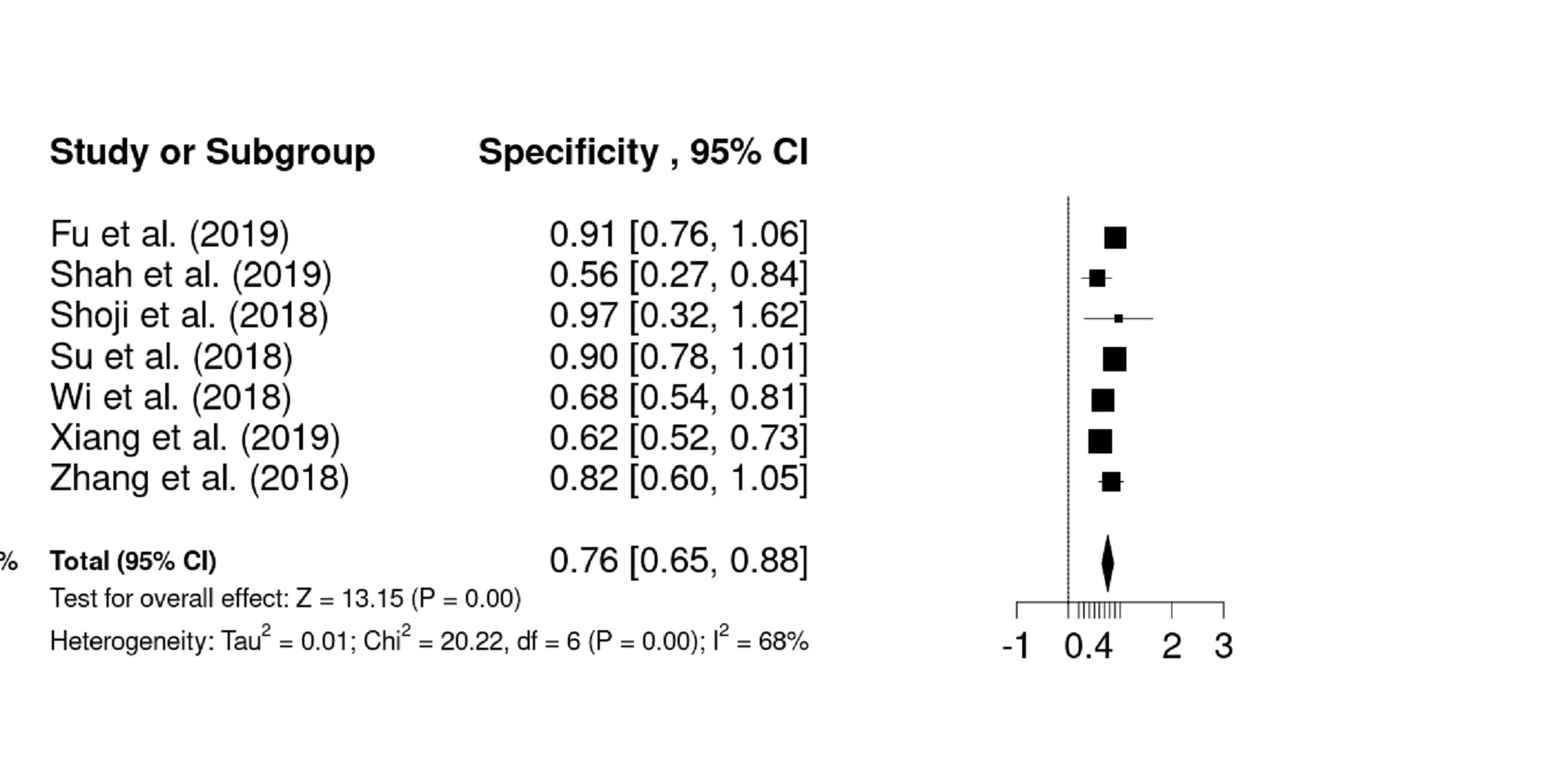

Supplement: Supplementary file 1 [file cancers-15-04105-s001.zip › Figure S8 [30,47-51].jpg]

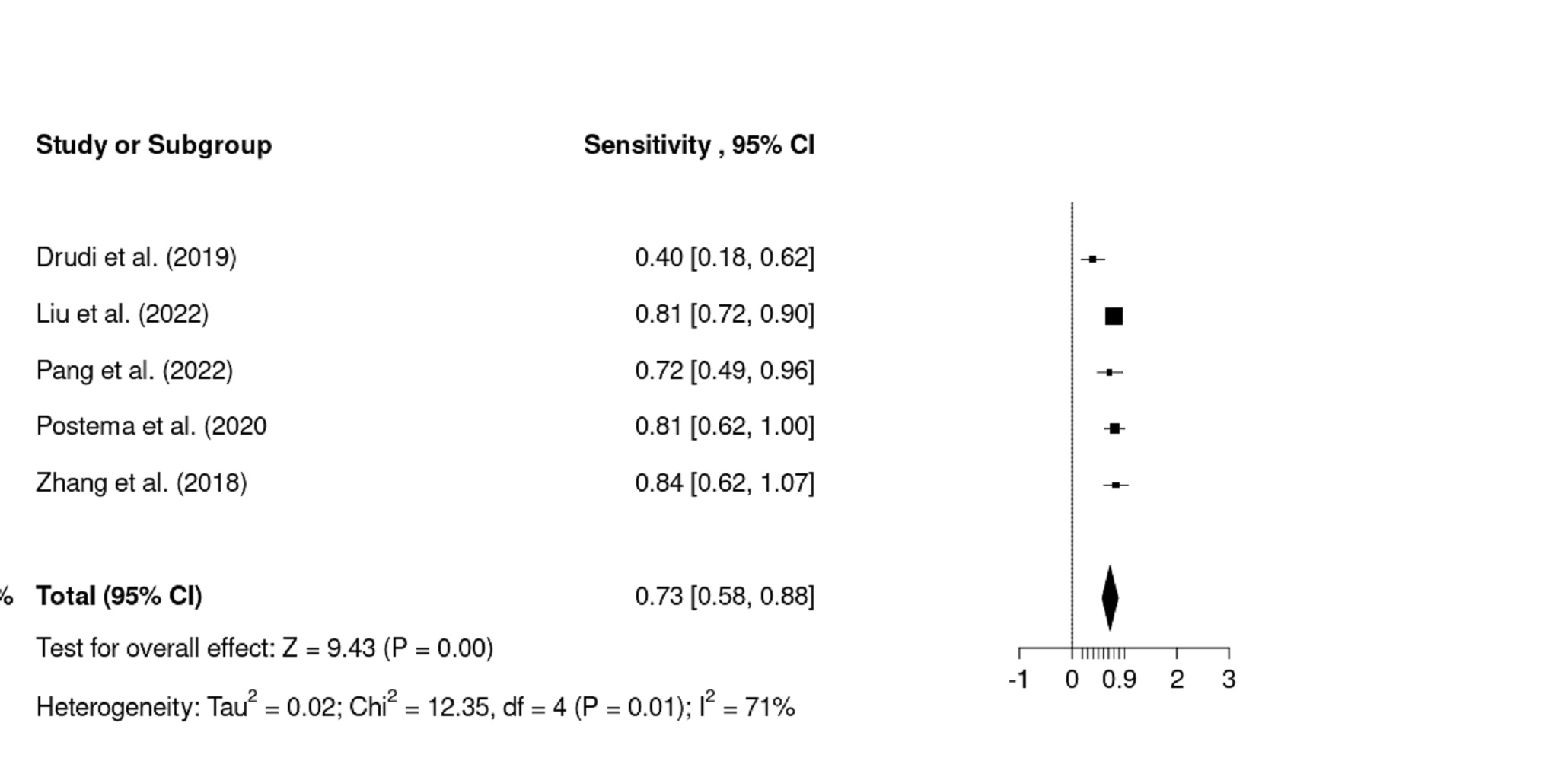

Supplement: Supplementary file 1 [file cancers-15-04105-s001.zip › Figure S9 [9,30,52-54].jpg]
